# The cyclin D1 proto-oncogene is sequestered in the cytoplasm of mammalian cancer cell lines

**DOI:** 10.1186/1476-4598-5-7

**Published:** 2006-02-17

**Authors:** John P Alao, Simon C Gamble, Alexandra V Stavropoulou, Karen M Pomeranz, Eric W-F Lam, R Charles Coombes, David M Vigushin

**Affiliations:** 1 Department of Cancer Medicine, Cancer Cell Biology Section, Imperial College, Hammersmith Hospital, Du Cane Road, London, W12 0NN, UK

## Abstract

**Background:**

The cyclin D1 proto-oncogene is an important regulator of G1 to S-phase transition and an important cofactor for several transcription factors in numerous cell types. Studies on neonatal cardiomyocytes and postmitotic neurons indicate that the activity of cyclin D1 may be regulated through its cytoplasmic sequestration. We have demonstrated previously, that TSA induces the ubiquitin-dependent degradation of cyclin D1 in MCF-7 breast cancer cells. Additional studies were initiated in order to further investigate the effect of TSA on cyclin D1 regulation using sub-cellular fractionation techniques.

**Results:**

Our studies revealed cyclin D1 to be localized predominantly within the cytoplasmic fraction of all cell lines tested. These observations were confirmed by confocal microscopy. GSK3β was found to be localized within both the nucleus and cytoplasm throughout the cell cycle. Inhibition of GSK3β or CRM1-dependent nuclear export resulted in only modest nuclear accumulation, suggesting that the cytoplasmic localization of cyclin D1 results from the inhibition of its nuclear import.

**Conclusion:**

We have shown by several different experimental approaches, that cyclin D1 is in fact a predominantly cytoplasmic protein in mammalian cancer cell lines. Recent studies have shown that the cytoplasmic sequestration of cyclin D1 prevents apoptosis in neuronal cells. Our results suggest that cytoplasmic sequestration may additionally serve to regulate cyclin D1 activity in mammalian cancer cells.

## Background

The cyclin D1 proto-oncogene is an important regulator of G1 to S-phase transition in numerous cell types from diverse tissues. Binding of cyclin D1 to its kinase partners, the cyclin dependent kinases 4 and 6 (CDK4\6) results in the formation of active complexes that phosphorylate the Retinoblastoma tumor suppressor protein (RB). Hyperphosphorylation of RB results in the release of RB-sequesterd E2F transcription factors and the subsequent expression of genes required for entry into S-phase. More recently, cyclin D1 has also been shown to act a cofactor for several transcription factors. Initial studies indicated that cyclin D1 is localized predominantly in the nucleus of asynchronously growing cells [1]. During cell cycle progression, protein levels of the cyclin begin to rise early in G1, prior to its rapid nuclear export and degradation within the cytoplasm. Interestingly, the nuclear export and\or degradation of cyclin D1 is required for S-phase progression as failure to remove the cyclin results in G1 arrest [1,2].

The nuclear export of cyclin D1 has been shown to require prior phosphorylation on Thr-286 by glycogen synthase kinase 3β (GSK3β) [3]. This phosphorylation of cyclin D1 was initially thought to regulate its ubiquitin-dependent degradation. Indeed, mutation of Thr-286 to alanine resulted in increased stability of the cyclin. Subsequent studies however, demonstrated that cyclin D1 ubiquitylation and its rapid degradation can occur independently of GSK3β under certain conditions [4,5]. Nevertheless, it is still generally believed that cyclin D1 accumulates within the nucleus during G1, and at the G1-S-phase transition, GSK3β accumulates in the nucleus and mediates phosphorylation, nuclear export and subsequent ubiquitin-dependent degradation of cyclin D1 in the cytoplasm. More recently, the serine/threonine kinase Mirk/Dyrk1B was shown to enhance cyclin D1 degradation by phosphorylating Thr288. Mirk activity is restricted to the G0-/early G1-phase of the cell cycle and may not regulate cyclin D1 in actively cycling cells [6].

Cyclin D1 has also been shown to be an important cofactor for several transcription factors independently of its CDK activity (reviewed in [7,8]). It is not understood however, how cells integrate the CDK-dependent and independent activities of cyclin D1. Cyclin D1 has been shown to be sequestered in the cytoplasm of neonatal but not fetal cardiomyocytes [9]. Studies on postmitotic neurons also indicate that the activity of cyclin D1 may be regulated through its cytoplasmic sequestration [10]. In these cells, the enforced nuclear localization of cyclin D1 induced apoptosis. The subcellular localization of cyclin D1 may thus play a role in regulating cellular survival.

The Histone Deacetylase Inhibitor (HDACI) Trichostatin A (TSA) has been shown to be able to induce cell cycle arrest, but the exact molecular mechanism involved is not clear. We have demonstrated previously, that TSA specifically induces the rapid ubiquitin-dependent degradation of cyclin D1 in MCF-7 breast cancer cells [11]. Treatment with TSA also appeared to induce the nuclear exclusion of wild type but not a Thr-286 mutant GFP-Cyclin D1 in MCF-7 cells. Additional studies were initiated in order to further investigate the effect of TSA on cyclin D1 using sub-cellular fractionation techniques. Interestingly, these studies revealed cyclin D1 to be localized predominantly within the cytoplasm of all cell lines and murine tissue samples examined. Further studies using confocal microscopy confirmed the predominantly cytoplasmic localization of cyclin D1. GSK3β was found to be localized within both the nucleus and cytoplasm throughout the cell cycle, suggesting that the nuclear export of cyclin D1 is a constitutive process. Inhibition of GSK3β or CRM1-dependent nuclear export resulted in only modest nuclear accumulation, suggesting that the cytoplasmic localization of cyclin D1 results from the inhibition of its nuclear import. Our results suggest that the regulation of cyclin D1 activity by cytoplasmic sequestration may be a common feature in transformed mammalian cell lines.

## Results

### Determination of cyclin D1 localization by sub-cellular fractionation

**Figure 1:**
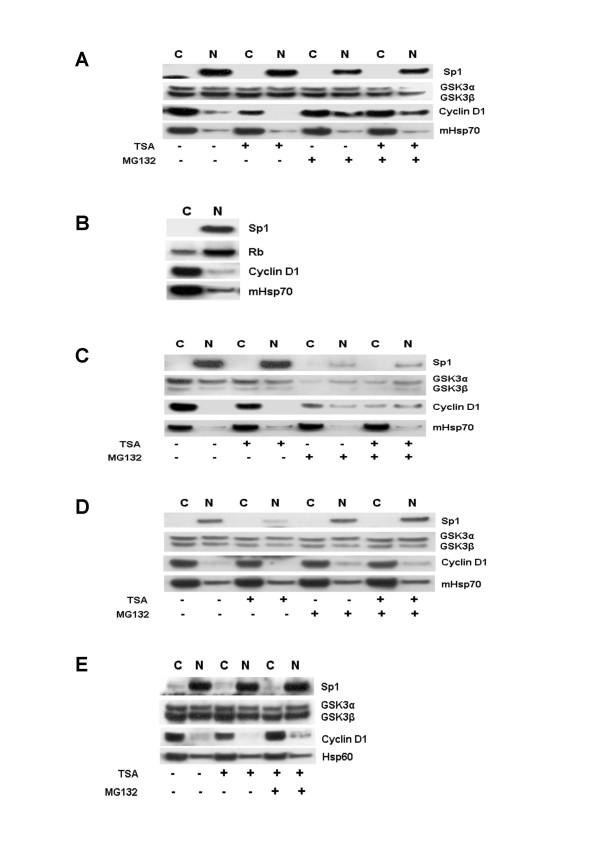
Effect of TSA on cyclin D1 localization and postranslational regulation. **A**. Asynchronously growing MCF-7 cells were treated with TSA (1 μM) and MG132 (50 μM) alone or in combination and subjected to subcellular fractionation as described in Methods. Cell lysates were separated by 4–20 % SDS-PAGE and immunoblot analysis was done using antibodies against Sp1, GSK3 and cyclin D1. Immunoblots against mitochondrial Sp1 and Hsp70 (mHsp70) served to ensure efficient subcellular fractionation and mHsp70 also served to monitor equal gel loading. **B. **Asynchronously growing MCF-7 cells were subjected to subcellular fractionation as described in A. Cell lysates were separated by 4–20 % SDS-PAGE and immunoblot analysis was done using antibodies against Sp1, Rb, cyclin D1 and mHsp70. **C. **Asynchronously growing MDA-MB231 were treated as in A. **D. **Asynchronously growing SKUT-1B cells were treated as in A. **E. **Asynchronously growing rat KNRK cells were treated as in A. Hsp60 served to ensure efficient subcellular fractionation and also served to monitor equal gel loading.

We have previously shown that TSA induces cyclin D1 degradation and the apparent nuclear exclusion of ectopically expressed GFP-cyclin D1 in human breast cancer cell lines. These results coupled with previous findings suggested that TSA induces cyclin D1 degradation through mediating its export to the cytoplasm. To test this idea, we determined by sub-cellular fractionation, the effect of TSA on endogenous cyclin D1 localization in asynchronous human cancer cells. Surprisingly, we observed the majority of cyclin D1 is detected in the cytoplasmic fraction of untreated, asynchronous exponentially growing MCF-7 and MDA-MB231 breast cancer cells (Figures 1A and 1B). In contrast, the levels of GSK3α and GSK3β were similar in both the cytoplasmic and nuclear fractions. We confirmed efficient cytoplasmic and nuclear fractionation by localization of Sp1 and Hsp60 (or mitochondrial Hsp70 [mHsp70]) that localized predominantly to the nucleus and cytoplasm respectively. Culture of MCF-7 breast cancer cells with 1 μM TSA for 6 h resulted in a reduction of cyclin D1 level within both fractions, while treatment with 50 μM MG132 (a proteasome and cathepsin inhibitor) resulted in increased levels of the cyclin D1 within the nuclear fraction. We also observed in these studies, that MG132 abolished the TSA induced loss of cyclin D1 in both fractions but did not affect the level or localization of GSK3α and GSK3β (Figure 1A). In contrast, retinoblastoma protein (pRb), the cyclin D1-CDK4/6 substrate was detected largely within the nuclear fraction (Figure 1B). Similar results were observed in MDA-MB231 cells, with the exception that co-culture with MG132 also resulted in reduced GSK3α and GSK3β levels within both fractions (Figure 1C).

**Figure 2:**
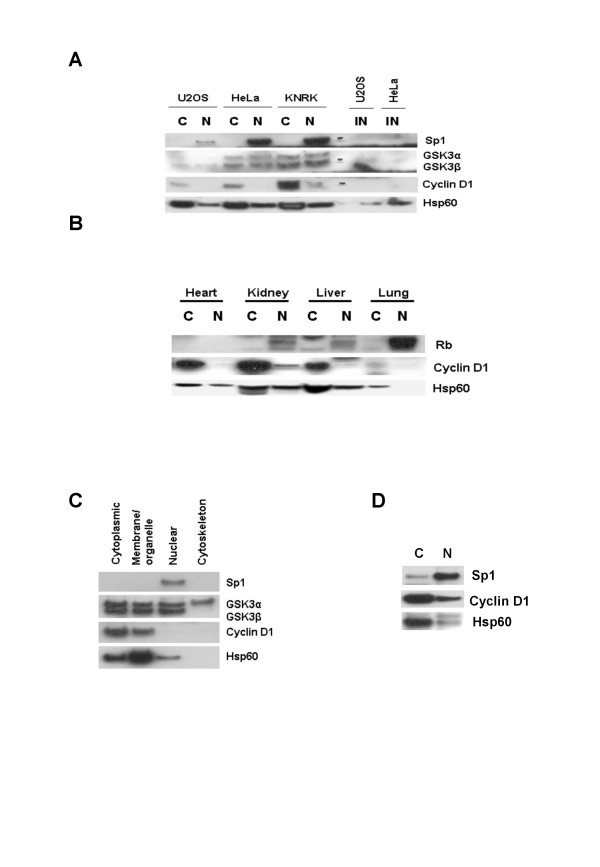
Cyclin D1 localization in human cancer cells and murine tissues. **A. **Asynchronously growing U2OS, HeLa and KNRK cells were subjected to subcellular fractionation as described in Methods. Cell lysates and insoluble fractions were separated by 4–20 % SDS-PAGE and immunoblot analysis was done using antibodies against Sp1, GSK3 and cyclin D1. **B. **Indicated mouse tissues were subjected to subcellular fractionation as described in the Methods section. Cell lysates were separated by 4–20 % SDS-PAGE and immunoblot analysis was done using antibodies against cyclin D1. Immunoblots against Rb and Hsp60 served to ensure efficient subcellular fractionation. **C. **Asynchronously growing MCF-7 cells were subjected to proteomic fractionation. Cell lysates were separated by 4–20 % SDS-PAGE and immunoblot analysis was done using antibodies against Sp1, GSK3 and cyclin D1. Immunoblots against Sp1 and Hsp60 served to ensure efficient subcellular fractionation. **D. **Asynchronously growing MCF-7 cells were subjected to subcellular fractionation using an in-house protocol. Cell lysates were separated by 4–20 % SDS-PAGE and immunoblot analysis was done using antibodies against Sp1, GSK3 and cyclin D1.

We also wished to determine if similar results could be observed in non-breast cancer cell lines and tissues. Firstly, we performed similar experiments in the SK-UT-1B uterine cancer cell line that has been shown previously to be defective in cyclin D1 degradation [12,13]. As expected, TSA treatment had little effect on cyclin D1 levels [11] although co-culture with MG132 did result in a slight increase in cyclin D1 accumulation within the nuclear fraction. This observation suggested that low levels of cyclin D1 degradation do occur in this cell line (Figure 1D). The results observed in KNRK transformed rat kidney fibroblasts were similar to those observed in MDA-MB231 cells (Figure 1E), with the exception that MG132 that a more pronounced effect on cyclin D1 stability. Additional experiments in U2OS osteosarcoma and HeLa cervical cancer cells indicated that cyclin D1 was also localized predominantly within the cytoplasmic fraction of these cells. In addition, cyclin D1 was not detected in the insoluble fraction obtained from these cells following the separation of the cytoplasmic and nuclear fractions (Figure 2A). Analysis of the cytoplasmic and nuclear fractions from murine heart, kidney, liver and lung tissue, similarly demonstrated that the highest levels of cyclin D1 were detectable within the cytoplasmic fraction (Figure 2B). These observations are in agreement with those from a recent study by De Falco *et al*., on cyclin D1 localization in murine tissues [14].

In order to verify these results, we used two additional protocols to obtain cytoplasmic and nuclear fractions from MCF-7 cells. Firstly, a second commercially available kit was used to sequentially isolate cytoplasmic, membrane/organelle, nuclear and cytoskeletal fractions. In these fractions, the highest levels of cyclin D1 were again detected in the cytoplasmic fractions and to a lesser extent, in the membrane/organelle fractions (Figure 2C). Sp1 was detected solely in the nuclear fraction while equivalent levels of GSK3α and GSK3β levels were detected in the cytoplasmic, membrane/organelle and nuclear fractions. Only GSK3α was detectable in the cytoskeletal fraction, while the highest levels of Hsp60 were detected in the cytoplasmic and membrane/organelle fractions. Lastly, we used an in-house protocol to prepare cytoplasmic and nuclear fractions from MCF-7 cells. In these experiments, the highest levels of cyclin D1 were again detected within the cytoplasmic fraction (Figure 2D). Our data thus strongly suggested that cyclin D1 is localized predominantly within the cytoplasm, at least in the various cell lines and tissues tested.

### Effect of cell cycle phase on cyclin D1 levels and sub-cellular localization

**Figure 3:**
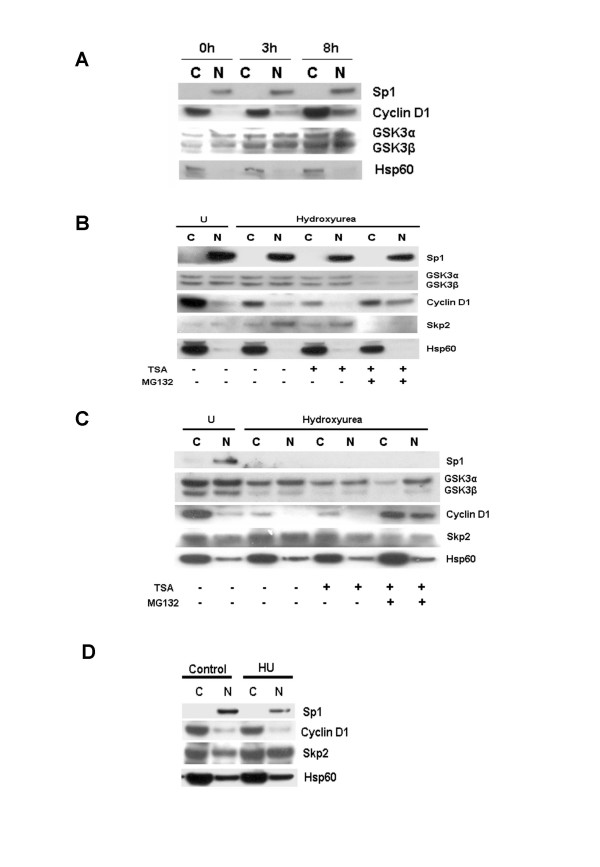
Effect of cell cycle progression on cyclin D1 localization. **A. **MCF-7 cells were cultured for 48 h in double stripped phenol red-free medium containing 100 nm ICI182,780. Cells were treated with fresh medium containing β17-oestrodiol (100 nm) and harvested at the indicated time points. Cell lysates were separated by 4–20 % SDS-PAGE and immunoblot analysis was done using antibodies against Sp1, GSK3, cyclin D1 and Hsp60. **B. **MCF-7 cells were left untreated or treated with 1 mM hydroxyurea (HU) for 24 h. HU treated cells were treated with TSA (1 μM) alone or with MG132 (50 μM) for 6 h and treated as in figure 1A. Immunoblot analysis was done using antibodies against Sp1, GSK3, Skp2, cyclin D1 and Hsp60. **C. **MDA-MB231 cells were treated as in B. **D. **SKUT-1B cells were treated for 24 h with HU (1 mM) and cell lysates separated by 4–20 % SDS-PAGE. Immunoblot analysis was done using antibodies against Sp1, Skp2, cyclin D1 and Hsp60.

The treatment of MCF-7 cells with the pure antiestrogen ICI182,780 (100 nM) for 48 h results in reduced cyclin D1 levels and G1 cell cycle arrest in this cell line [15-17]. Treatment of these cells with 17β-oestrodiol following the removal of ICI182,780, thus results in cell cycle progression that is accompanied by and dependent on the increased expression of cyclin D1. We employed this system in order to investigate the effect of 17β-oestrodiol on cyclin D1 accumulation and localization in cytoplasmic and nuclear fractions from MCF-7 cells. Increased levels of cyclin D1 were detectable in both fractions at 3 h and were maximal at ~8 h as previously reported [17]. In our system however, the highest levels of cyclin D1 were again detectable within the cytoplasmic fraction, irrespective of the time point (Figure 3A).

We next examined the effect of cell cycle arrest at the G1/S-phase boundary on cyclin D1 localization and levels in MCF-7, MDA-MB231 and SK-UT-1B cells. Cells were left untreated or incubated in the presence of 1 mM hydroxyurea for 24 h. Under these conditions, >90% of hydroxyurea treated cells arrested in late G1 or early S-phase (results not shown). We observed a marked reduction in cyclin D1 levels in MCF-7 and MDA-MB231 treated cells (Figures 3B and 3C). Reduced cyclin D1 levels were accompanied by increased nuclear levels of the Skp2 F-box protein that has been previously linked to the ubiquitin-dependent degradation of this cyclin [12,18]. The degree of cyclin D1 downregulation was clearly proportional to the increase in Skp2 levels (compare Figures 3B and 3C). The co-culture of hydroxyurea pre-treated cells with 1 μM TSA for 6 h, lead to further reductions in cyclin D1 levels in MCF-7 but not MDA-MB231 cells. Co-culture of both cell lines with TSA and MG132 (50 μM) however, lead to increased levels of cyclin D1 within both the cytoplasmic and nuclear fractions of both cell lines (Figures 3B and 3C). This observation suggested that the rate of cyclin D1 degradation is increased in both cell lines under these conditions. Hydroxyurea did not affect the levels or localization of GSK3α and GSK3β in MCF-7 cells but induced the downregulation of GSK3β in MDA-MB231 cells. Interestingly, co-culture with hydroxyurea did not result in a significant reduction of cyclin D1 levels in SK-UT-1B cells, despite a rise in nuclear Skp2 levels (Figure 3D). Since the reduction of nuclear cyclin D1 levels has been reported to be a prerequisite for S-phase progression, our observations raise interesting questions about how these cells might progress into S-phase in the absence of cyclin D1 degradation.

### Analysis of cyclin D1 localization by confocal microscopy

**Figure 4:**
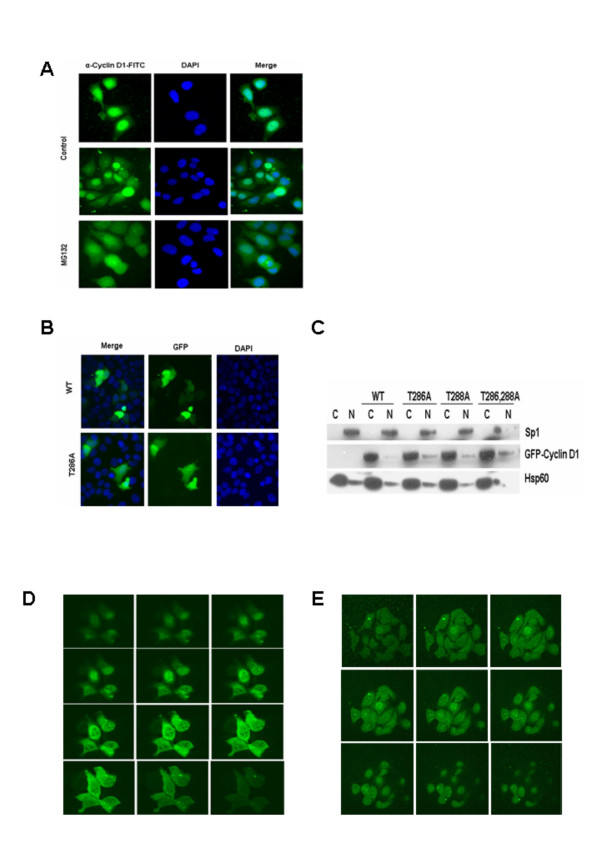
Determination of cyclin D1 localization by confocal microscopy. **A. **MCF-7 cells were allowed to attach to coverslips overnight and were then treated with or without MG132 (50 uM) for 6 h. Cells were fixed in ice cold methanol, washed and stained overnight with an FITC conjugated anti- cyclin D1 antibody, counterstained with DAPI and examined by fluorescence microscopy. **B. **MCF-7 cells grown on coverslips were transfected with wild type or T286A mutant GFP-cyclin D1 and incubated overnight to allow for expression of the recombinant protein. Cells were fixed in ice cold methanol, counterstained with DAPI and examined by direct fluorescence microscopy. **C. **MCF-7 cells were left untransfected or transfected with the indicated GFP-cyclin D1 constructs. Cell lysates were separated by 4–20 % SDS-PAGE. Immunoblot analysis was done using antibodies against Sp1, GFP and Hsp60. **D. **MCF-7 cells were grown on coverslips and transfected with wild type GFP-cyclin D1. Cells were fixed in ice cold methanol and examined by confocal microscopy. **E. **MCF-7 cells were allowed to attach to coverslips overnight and were then fixed in ice cold methanol, washed and stained overnight with an FITC conjugated anti- cyclin D1 antibody. Cells were subsequently examined by confocal microscopy.

Based on immunofluorescent studies, cyclin D1 has been previously localized to the nucleus of asynchronously growing cells [1]. Our observation that cyclin D1 localization was mainly restricted to the cytoplasmic fraction of the cell lines and tissues examined in this study was therefore surprising. Indeed, experiments from our own laboratory using an FITC-conjugated anti-cyclin D1 antibody also suggested a predominantly nuclear localization for this cyclin (Figure 4A). We observed however, that in MCF-7 cells transiently expressing GFP-Cyclin D1, the recombinant protein appeared to be localized to both the cytoplasm and nucleus when examined by direct immunofluorescence microscopy (Figure 4B). When these cells were subjected to sub-cellular fractionation and analysed by immunoblot analysis with a monoclonal antibody against GFP, the recombinant protein was again detected predominantly within the cytoplasmic fraction (Figure 4C). Single or combined mutations of Thr-286 or Thr-288 to alanine resulted in only a slight increase of the recombinant protein within the nuclear fraction. These observations strongly suggested that the apparent nuclear localization of cyclin D1 as determined by indirect immunofluorescence microscopy may be an artefact of this particular technique. We thus decided to examine the localization of both endogenous and transiently expressed GFP-Cyclin D1 in MCF-7 cells by confocal microscopy. These experiments confirmed that cyclin D1 does not indeed, localize predominantly within the nucleus of mammalian cells (Figures 4D and 4E).

### Combined proteasome and CRM1 inhibition does not result in the nuclear localization of cyclin D1

**Figure 5:**
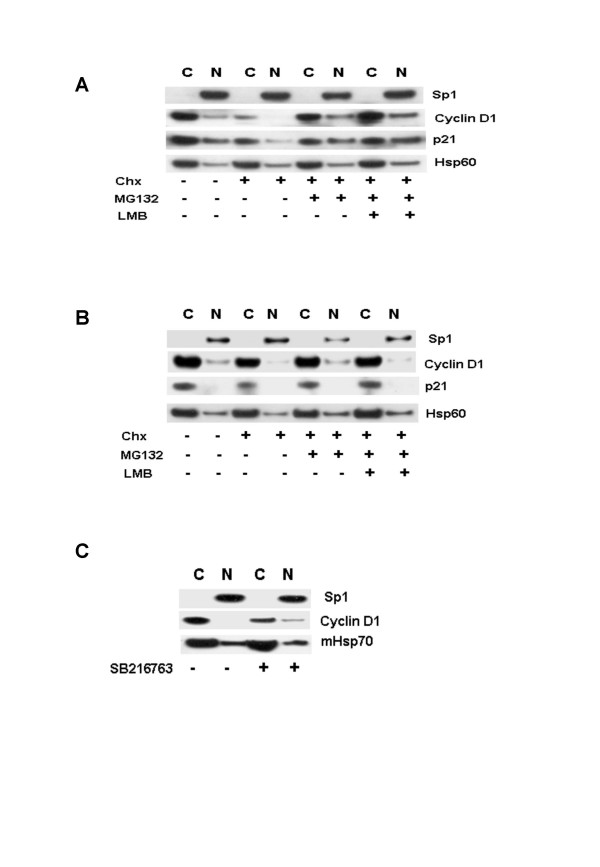
Cytoplasmic sequestration of cyclin D1 in human cancer cell lines. **A. **Asynchronously growing MCF-7 cells were treated with cycloheximide (Chx) (50 μM) alone and together with MG132 (50 μM) or LMB 10 ng/ml as indicated. Cell lysates were separated by 4–20 % SDS-PAGE and immunoblot analysis was done using antibodies against Sp1, GSK3, cyclin D1, p21 and Hsp60. **B. **Asynchronously growing SKUT-1B cell were treated as in A. **C. **MCF-7 cells were treated for 24 h with the GSK3-specific inhibitor SB216763. Cell lysates were separated by 4–20 % SDS-PAGE and immunoblot analysis was done using antibodies against Sp1, cyclin D1 and mHsp70.

The inhibition of protein synthesis by cycloheximide results in rapid disappearance cyclin D1 in several cell lines [19]. According to the current model for cyclin D1 posttranslational regulation and our data presented above, cytoplasmic cyclin D1 would need to rapidly enter the nucleus, where its phosphorylation and subsequent ubiquitylation would target it for nuclear export and degradation within the cytoplasm [12,20]. We thus wished to determine, if we could induce the predominant nuclear localization of cyclin D1 in cycloheximide treated cells by simultaneously inhibiting nuclear export and degradation of the cyclin. Co-culture of asynchronous MCF-7 cell populations with 50 μM cycloheximide for 2 h resulted in the loss of cyclin D1 in both fractions (Figure 5A). This effect could be abolished by MG132 (50 μM), leading to the stabilization of the cyclin in both fractions. Co-culture of cycloheximide treated cells with MG132 and LMB (an inhibitor of CRM1), lead to further increases in the level of cyclin D1 within both fractions but not in the predominant nuclear localization of the protein. Similar treatments in SK-UT-1B cells did not affect the levels or localization of cyclin D1 (Figure 5B). Inhibition of GSK3 activity by SB216763 resulted in modest nuclear accumulation of cyclin D1 in MCF-7 cells (Figure 5C). Our data thus clearly demonstrates, that i) cyclin D1 localizes predominantly to the cytoplasm in all cell lines and tissues examined and ii) that the nuclear exclusion/cytoplasmic sequestration of the cyclin as opposed to its accumulation appears to be the rule. Cyclin D1 downregulation has been shown to be a prerequisite for S-phase progression [2]. Our findings thus offer a novel explanation for the continued proliferation of cells that are either defective in cyclin D1 downregulation [12,13], or express variants lacking the C-terminal Thr-286 residue required for nuclear export and degradation within the cytoplasm [21]. The failure of cyclin D1 to relocalize to the nucleus when both its degradation and nuclear export are inhibited, suggests that the nuclear import of this protein is inhibited in these cell lines.

## Discussion

Here we show that cyclin D1 is localized predominantly within the cytoplasm of mammalian cancer cells. Recent studies in our laboratory have demonstrated that TSA induces the rapid degradation of cyclin D1 in MCF-7 breast cancer cells and to a lesser extent in several other cell lines [11]. In these studies, TSA induced the apparent nuclear exclusion of wild type GFP-Cyclin D1 but not a Thr-286 mutant in MCF-7 cells transiently expressing these proteins. We used sub-cellular fractionation kits in order to determine if TSA would have a similar effect on the localization of endogenous cyclin D1. Early studies suggested that cyclin D1 localized predominantly to the nucleus of asynchronously growing embryonic lung fibroblasts [1]. Later studies in cardiomyocytes and neuronal cells demonstrated the predominantly cytoplasmic localization in these cells [9,10]. Interestingly, Tamamori-Adachi *et al*., [9] observed that although ectopically expressed cyclin D1 accumulated to a greater degree in fetal cardiomyocytes, this localization was not predominantly nuclear. The results presented here indicate that the highest levels of cyclin D1 are consistently detectable within the cytoplasmic fraction, irrespective of the cell cycle phase, or cell line examined. Furthermore, equal levels of GSK3β were detectable in both cytoplasmic and nuclear fractions throughout the cell cycle.

The mechanisms that regulate the nuclear exclusion of cyclin D1 remain unclear. Inhibition of CRM1-dependent nuclear export by LMB has been shown previously, to induce the nuclear localization of cyclin D1 in mouse embryonic fibroblasts [20]. Our results show however, that the inhibition of GSK3β or CRM1-dependent nuclear export does not result in the effective nuclear accumulation of cyclin D1 in mammalian cancer cell lines. In our experiments, inhibition of GSK3β or CRM1 activity resulted in the modest nuclear accumulation of cyclin D1. GSK3β may thus play a role in regulating the nuclear levels of cyclin D1. When G1 arrested MCF-7 cells were induced to re-enter the cell cycle, cyclin D1 accumulated mainly within the cytoplasmic fraction and to a lesser extent within the nuclear fraction. In addition, the combined inhibition of proteasomal degradation and nuclear export inhibition in cycloheximide treated cells resulted in only a modest nuclear accumulation of cyclin D1 (Figure 5). These findings suggest that the impaired nuclear import of cyclin D1 results in its cytoplasmic sequestration in these cells lines.

In our hands, indirect immunofluorescence studies with an FITC-conjugated anti-cyclin D1 antibody suggested a predominantly nuclear localization for the cyclin. This antibody belongs to the same clone that suggests a predominantly cytoplasmic localization of cyclin D1 in immunoblot analyses of fractionated cell lysates. In contrast, transiently expressed wild type or Thr-286 mutated GFP-Cyclin D1 exhibited an even nucleo-cytoplasmic localization when examined by direct fluorescence microscopy. Westernblotting analyses of cytoplasmic and nuclear extracts however, indicated that both the endogenous and recombinant proteins are localized predominantly within the cytoplasm. Confocal microscopy studies on cells expressing GFP-Cyclin D1 or endogenous cyclin D1 confirmed the predominantly cytoplasmic localization of these proteins. Our observations suggest that the apparent nuclear localization of cyclin D1 (as determined by indirect immunofluorescence microscopy) is an artefact of the particular technique.

## Conclusion

Early studies on cyclin D1 localization have been based on indirect immunofluorescence studies in embryonic. We have shown by several different experimental approaches, that cyclin D1 is in fact a predominantly cytoplasmic protein in mammalian cancer cell lines. Our findings also indicate that the cytoplasmic sequestration of cyclin D1 may result from the inhibition of its nuclear import. The important role that cyclin D1 plays in both the normal and pathological setting has thus ensured that this protein remains the active focus of several research groups. Therefore, the development of accurate models to describe the regulation of cyclin D1 under both normal and pathological conditions is not only highly desirable but extremely important. Our findings provide evidence for the regulation of cyclin D1 activity by cytoplasmic sequestration in several mammalian cancer cell lines and murine tissues. Recent studies have shown that the cytoplasmic sequestration of cyclin D1 prevents apoptosis in neuronal cells [10,22,23]. Our results suggest that cytoplasmic sequestration may additionally serve to regulate cyclin D1 activity in mammalian cancer cells.

## Methods

### Reagents

Carbobenzoxy-leucyl-leucyl-leucinal (Z-LLL-CHO, MG132) (Calbiochem, VWR International Ltd., Lutterworth, United Kingdom) was dissolved in DMSO at the indicated concentrations and stored at -20°C. Stock solutions of TSA, 17β-oestrodiol and cycloheximide in ethanol and leptomycin B in 70% (v/v) methanol (Sigma-Aldrich; Dorset, united Kingdom) were stored at -20°C. The GSK3-specific inhibitor SB216763 (Tocris Bioscience, Avonmouth, United Kingdom) was dissolved in DMSO and stored at -20°C. Antibodies to cyclin D1, actin, Sp1, Skp2, p21 and GFP (Santa Cruz Biotechnology, santa Cruz, CA), GSK3 (Upstate Biotechnology, Dundee, United Kingdom), Hsp60, mHsp70 (Abcam, Cambridge, United Kingdom), and β-Catenin (Transduction Laboratories, BD Biosciences Ltd., Oxford, United Kingdom, Santa Cruz Biotechnology) were used.

### Cell culture

MCF-7, MDa-MB231, HeLa, SK-UT-1B cells, KNRK and U2OS cells (American Type Culture Collection, Rockville, MD) were cultured in DMEM supplemented with 10% (v/v) fetal calf serum, 2 mM L-glutamine, 100 units/ml penicillin and 100 μg/ml streptomycin at 37°C in humidified 5% CO_2_.

### Sub-cellular fractionation

Cytoplasmic and nuclear fractions were prepared from asynchronously growing cells or fresh murine tissue samples using an NE-PER^® ^Nuclear-cytoplasmic kit (Perbio, Erembodegem, Belgium) according to the manufacturer's instructions. Alternatively, cytoplasmic, membrane/organelle, nuclear and cytoskeletal fractions were prepared using a ProteoExtract^® ^Subcellular Proteome Extraction Kit (Calbiochem, VWR International Ltd., Lutterworth, United Kingdom) according to the manufacturer's instructions. The purity of each fraction was analyzed by immunoblotting using antibodies against Sp1 and Hsp60 (or mitochondrial Hsp70 [mHsp70]).

### Transfection and immunoblot analysis

Fugene 6 transfection reagent (Roche Diagnostics Ltd, East Sussex, United Kingdom) was used for DNA plasmind transfection. asynchronous cell populations at a density of 50–60% in 6-well plates or on coverslips were transfected with 1–2 μg of plasmid DNA, following the formation of lipid-DNA complexes for 20 min at room temperature in Optimem I medium (Invitrogen Ltd., Paisley, United Kingdom). Complexes were added directly to cells growing in 2 ml DMEM and incubated for 5 h followed by washing with PBS buffer and the addition of fresh medium. Cells were used 24 h after transfection and the recombinant proteins detected by immunoblotting, or by fluorescence microscopy as previously described [11].

### Confocal microscopy

MCF-7 cells were grown on sterile glass coverslips in 24-well plates to 30% confluence in RPMI media before being washed three times in PBS. Cells were fixed in methanol at -20°C for ten minutes. Coverslips were washed a further three times in PBS and treated with 10% whole goat serum (Santa Cruz) for thirty minutes. Antibodies to FITC-conjugated anti-Cyclin D1 (Santa Cruz) (1:50 dilution) were applied in L15 (Sigma-Aldrich) medium overnight. Cells were washed five times in PBS, mounted on glass slides with Vectorshield containing DAPI (Vector Laboratories Inc.) and visualised on a Zeiss Meta 512 confocal microscope.

## Abbreviations

TSA- trichostatin A, Z-LLL-CHO- carbobenzoxy-leucyl-leucyl-leucinal (MG132), LMB- Leptomycin B, DMSO- dimethyl sulphoxide, DMEM- Dulbecco's modified eagle medium, DAPI- 4', 6'- diamidino-2-phenylindole dihydrochloride, PBS- phosphate buffered saline, RT-PCR- reverse transcription- polymerase chain reaction, FITC- fluorescein isothiocyanate, GSK3α- glycogen synthase 3 alpha, GSK3β- glycogen synthase 3 beta, GFP- Green fluorescent protein, Hsp60- Heat shock protein 60, mHsp70- mitochondrial Heat shock protein 70, Rb- retinoblastoma protein

## Competing interests

The author(s) declare that they have no competing interests.

## Authors' contributions

JPA, DMV, EW-FL and RCC conceived of the study, coordinated its design and execution and drafted the manuscript. JPA carried out subcellular fractionation, immunoblotting analyses and immunofluorescence microscopy. AVS carried out cell culture and immunoblot experiments. SCG carried out confocal microscopy experiments. KMP carried out nuclear-cytoplasmic fractionation. JPA, SCG, RCC, and EW-FL interpreted and analyzed the data. All authors read and approved the final draft manuscript.
